# Oncogenic Potential of the Dual-Function Protein MEX3A

**DOI:** 10.3390/biology10050415

**Published:** 2021-05-07

**Authors:** Marcell Lederer, Simon Müller, Markus Glaß, Nadine Bley, Christian Ihling, Andrea Sinz, Stefan Hüttelmaier

**Affiliations:** 1 Charles Tanford Protein Center, Faculty of Medicine, Institute of Molecular Medicine, Section for Molecular Cell Biology, Martin Luther University Halle-Wittenberg, Kurt-Mothes-Str. 3a, 06120 Halle, Germany; simon.mueller@medizin.uni-halle.de (S.M.).; markus.glass@medizin.uni-halle.de (M.G.).; nadine.stoehr@medizin.uni-halle.de (N.B.); stefan.huettelmaier@medizin.uni-halle.de (S.H.); 2 Center for Structural Mass Spectrometry, Department of Pharmaceutical Chemistry & Bioanalytics, Institute of Pharmacy, Martin Luther University Halle-Wittenberg, Kurt-Mothes-Str. 3, 06120 Halle (Saale), Germany; christian.ihling@pharmazie.uni-halle.de (C.I.); andrea.sinz@pharmazie.uni-halle.de (A.S.)

**Keywords:** MEX3A, oncofetal, cancer, KH domain, RNA binding, RING domain, ubiquitination

## Abstract

**Simple Summary:**

RNA-binding proteins (RBPs) are involved in the post-transcriptional control of gene expression, modulating the splicing, turnover, subcellular sorting and translation of (m)RNAs. Dysregulation of RBPs, for instance, by deregulated expression in cancer, disturbs key cellular processes such as proliferation, cell cycle progression or migration. Accordingly, RBPs contribute to tumorigenesis. Members of the human MEX3 protein family harbor RNA-binding capacity and E3 ligase activity. Thus, they presumably combine post-transcriptional and post-translational regulatory mechanisms. In this review, we discuss recent studies to emphasize emerging evidence for a pivotal role of the MEX3 protein family, in particular MEX3A, in human cancer.

**Abstract:**

MEX3A belongs to the MEX3 (Muscle EXcess) protein family consisting of four members (MEX3A-D) in humans. Characteristic for MEX3 proteins is their domain structure with 2 HNRNPK homology (KH) domains mediating RNA binding and a C-terminal really interesting new gene (RING) domain that harbors E3 ligase function. In agreement with their domain composition, MEX3 proteins were reported to modulate both RNA fate and protein ubiquitination. MEX3 paralogs exhibit an oncofetal expression pattern, they are severely downregulated postnatally, and re-expression is observed in various malignancies. Enforced expression of MEX3 proteins in various cancers correlates with poor prognosis, emphasizing their oncogenic potential. The latter is supported by MEX3A’s impact on proliferation, self-renewal as well as migration of tumor cells in vitro and tumor growth in xenograft studies.

## 1. Introduction

Post-transcriptional control of gene expression influences a variety of cellular processes. In this context, RNA-binding proteins (RBPs) modulate gene expression in various multiprotein complexes via a plethora of different mechanisms, ranging from, e.g., alternative splicing, control of RNA turnover, regulation of mRNA translation, subcellular mRNA localization to post-transcriptional gene editing. In agreement with the versatile role in modulating the fate of genetic information, encompassing coding as well as non-coding RNAs (ncRNAs), deregulation of RBP expression and function severely impacts tumorigenesis. Consistent with cancer presenting a multi-pathway disease and multiple roles of RBPs in modulating gene expression, RBPs influence essentially all cancer hallmarks [1,2,3]. This highlights RBPs as promising therapeutic targets [4,5].

Progress in omic technologies revealed a plethora of canonical as well as novel noncanonical RBPs, unraveling a surprising diversity and plethora of mechanisms guiding the post-transcriptional fate of transcripts. Among 1542 identified RBPs reported in the RBP census [6], only about 350 proteins were categorized within the Gene Ontology molecular function “catalytic activity” [7]. Out of these, 32 RBPs contain validated or putative E3 ligase activity and include all four human members of the MEX3 protein family (Table 1).

## 2. MEX3 Proteins Link the Control of RNA Fate with Protein Ubiquitination

Initially, MEX3 was identified in *C. elegans* [8], where the protein was implicated in regulating the localized translation of pal-1 mRNA at the posterior of early embryos. The loss of MEX3 leads to ectopic Pal-1 expression resulting in an anterior muscle excess, which set the stage for the name of the protein family. In contrast to its mammalian orthologs, *C. elegans* MEX3 contains two KH domains mediating RNA binding but lacks a C-terminal RING domain.

**Figure 1:**
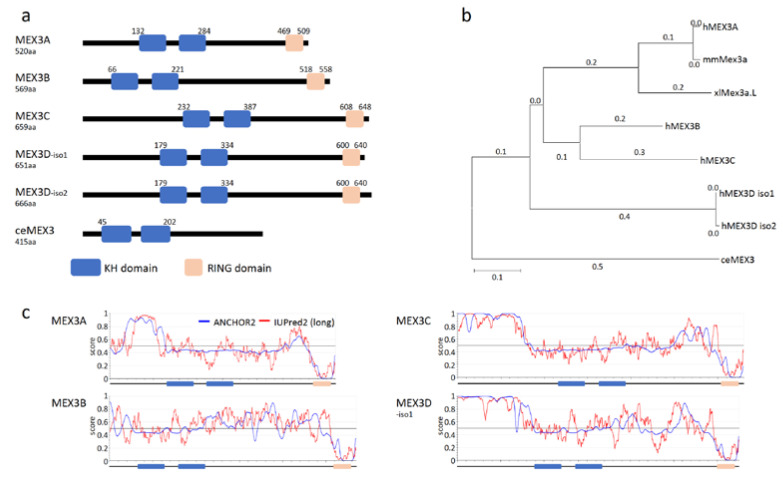
MEX3 family of RNA-binding proteins. (**a**) Domain structure of human MEX3 proteins and the founder ortholog ceMEX3 of *Caenorhabditis elegans* with RNA-binding domains (HNRNPK homology domains; KH; blue) and the really interesting new gene (RING domain; orange). Proteins shown: MEX3A (Acc. No. NM001093725.2), MEX3B (Acc. No. NM032246.6), MEX3C (Acc. No. NM016626.5), MEX3D-isoform1 (Acc. No. NM203304.4), MEX3D-isoform2 (Acc. No. NM001174118.2) and MEX3 (Acc. No. NM001381229.2). Annotation and localization of KH di- and RING domain according to www.uniprot.org (accessed on 26 March 2021) [11]. (**b**) A phylogenetic tree indicating amino acid substitutions of distinct MEX3 paralogs from different species (h: human, mm: *Mus musculus*, xl: *Xenopus laevis* and ce: *Caenorhabditis elegans*). Protein sequences were aligned using Tcoffee before creating a phylogenetic tree with MEGA X. (**c**) Prediction of intrinsically disordered regions (IUPred2; red) and disordered protein-binding regions (ANCHOR2; blue) using IUPred2A (https://iupred2a.elte.hu; accessed on 30 March 2021).

The human MEX3 protein family comprises four RNA-binding proteins (RBPs), termed MEX3A-D. In addition to two HNRNPK-homology (KH) domains involved in RNA-binding, human MEX3 proteins are characterized by a C-terminal RING (really interesting new gene) domain (Figure 1a; [9,10]). Recent studies revealed that MEX proteins, especially the so far best-studied paralogs (MEX3A, B and C), modulate oncogenic cell properties, including proliferation, migration, EMT (epithelial-to-mesenchymal-transition) and colony formation, suggesting roles in the regulation of tumor cell self-renewal potential. In agreement, members of the MEX3 protein family are expressed in a variety of cancers where their elevated expression is frequently associated with reduced overall survival probability.

Mammalian MEX3 proteins are predominantly cytoplasmic but reported to harbor nucleocytoplasmic shuttling activity due to their NES (nuclear export signal) and NLS (nuclear localization signal) motifs. This property suggests that MEX3 proteins may already associate with target transcripts in the nucleus. In the cytoplasm, MEX3A and B localize presumably in an RNA-dependent manner also to P-bodies, whereas MEX3C is evenly distributed in the cytoplasm [9]. P-bodies resemble RNP granules that are due to their composition presumably involved in mRNA turnover, translational control or miRNA-directed regulation of mRNA fate via the RISC complex (for review, see: Ref. [12]). Consistently, MEX3A and B interact in an RNA-dependent manner with Argonaute proteins Ago1 and Ago2. In contrast to MEX3A and B, MEX3C interacts with Ago1 and 2 in an RNA-independent manner [9]. This interaction with components of the RISC complex could delineate a possible mechanism of post-transcriptional gene regulation of this protein family. Additionally, MEX3A enables a noncanonical function of miR-126-5p in the cell nucleus. Ago2-bound miR-126-5p forms a complex with MEX3A on the surface of autophagic vesicles and is transported into the nucleus [13], where miR126-5p prevents caspase-3 dimerization and activation. This supports a dual compartment role, at least of MEX3A.

The canonical domain organization of the four mammalian MEX3 paralogs (54 to 69kDa) is highly conserved (Figure 1b). The overall sequence identity between the paralogs ranges between 37–42% (similarity 45–50%), with a higher degree of identity for the KH1/2 di-domain (78–91%) and the RING domain (68–85%). These similarities suggest similar biochemical functions in RNA binding and E3 ligase activity. Notably, however, the paralogs differ in the position of these functional domains. Whereas the RING domain is positioned at the very C-terminus of the proteins, the N-terminal part, as well as the spacing of the KH1/2-didomain and RING domain (sequence identity 21–39%; Figure S1), varies substantially between paralogs (Figure 1a). These quite divergent sequence regions could contribute to different cellular functions of the paralogs, mediated by a potential association with different factors, e.g., E3 ligase substrates or co-factors modulating MEX3 function, but so far, this hypothesis is not supported by published data. Notably, these regions contain intrinsically disordered stretches (Figure 1c) with disordered protein-binding regions according to IUPred2 and ANCHOR2 prediction [14].

All members of the MEX3 protein family bind RNA irrespective of the organism or cell type. Initial in vitro SELEX studies using the C. elegans MEX-3 KH1/2 di-domain as bait identified an (A/G/U)(G/U)AGN_0-8_U(U/A/C)UA MEX-3 Recognition Element (MRE) [15]. This was further evaluated by high-resolution protein/RNA co-crystal structures of the individual human MEX3C KH1 (PDB ID 5WWW) and KH2 (PDB ID 5WWX) domains [16]. However, these studies only provide a limited view on the putative role of the MEX3 KH di-domains in RNA binding and shaping of RNA-protein scaffolds for the assembly of larger complexes, as reported for other KH di-domain protein like IGF2BPs [17,18,19,20].

The role of the RING finger domain of MEX3 proteins has mainly been studied in the context of MEX3B and MEX3C. However, all human MEX3 proteins harbor a conserved C-Terminal C3HC4-type RING domain suggesting that all four proteins harbor E3 ligase activity. Crystal structure of MEX3C (PDB ID 5ZI6) in combination with superimposition models with the MDM2–MDMX–UbcH5b–Ub (PDB ID 5MNJ) complex led to the identification of critical residues for auto-ubiquitination as well as E2 ligase interaction [21].

So far, a variety of target mRNAs or proteins ubiquitinated by MEX3 proteins have been proposed. However, there is no comprehensive view on the RNA-binding properties of the protein family since neither CLIP (cross-linking immunoprecipitation) nor RIP (RNA immunopurification) studies have been reported to the best of our knowledge. Likewise, only a few E3 ubiquitin ligase substrates of human MEX3 proteins have been reported. This substantially limits our understanding of how MEX3 proteins may link the control of RNA fate and protein ubiquitination.

## 3. Ubiquitination Targets of MEX3 Proteins

The impact and functional interaction of MEX3 proteins’ domains, namely the RNA-binding KH1/2 di-domain and the RING domain, in tumor cells remain largely unknown. However, recent findings provide the first insight on the interconnection of RNA binding and E3 ligase function provided through MEX3 protein KH domains and RING domains (Table 2).

Ubiquitination of all three to date reported E3 ligase substrate proteins of MEX3B requires association with the lncRNA HOTAIR, serving as a scaffold for MEX3B-directed protein ubiquitination [23,24,25]. HOTAIR (Homeobox transcript antisense RNA) is one of the most prominent oncogenic lncRNAs affecting a variety of cancer hallmarks, e.g., tumor cell survival and self-renewal [30]. Although lncRNAs, in general, are involved in transcription and post-transcriptional modulation, an interaction between HOTAIR and RNA-binding proteins with E3 ubiquitin ligase domains (DZIP3 and MEX3B) implies a post-translational function [23]. HOTAIR scaffolds and thus induces ubiquitination and degradation of ATXN1 (by DZIP3) and SNUPN (mediated by MEX3B), preventing premature senescence [23]. HOTAIR also serves as a scaffold for MEX3B-mediated ubiquitination and degradation of the tumor-suppressive transcription factor RUNX3 [25] as well as the polycomb-repressive complex 2 (PRC2) component SUZ12 [24]. SUZ12 proteasomal degradation is induced by a hepatitis B viral infection as the RNA helicase DDX5 is replaced on HOTAIR by MEX3B. MEX3B, in turn, leads to ubiquitination and subsequent degradation of SUZ12, mediating the re-expression of genes repressed by the PRC2 complex [24].

Intriguingly, in contrast to MEX3B, the MEX3C mediated ubiquitination of so far identified target proteins (PTEN, CNOT7 and RIG-I) does not induce their degradation but alters their activity. K27-linked poly-ubiquitination of PTEN (PTENK27-polyUB) by MEX3C occurs also in a lncRNA-dependent manner [26]. Various stimuli, including glucose or TGF-ß exposure, induce the activation of the GAEA (Glucose Aroused for EMT Activation) lncRNA. Upon glucose stimulation, GAEA and the E2 Ligase UBE2S associate with MEX3C resulting in poly-ubiquitination of PTEN. PTENK27-polyUB dephosphorylates TWIST1, SNAI1 and YAP1, leading to the accumulation of these EMT (epithelial-to-mesenchymal transition)-drivers. In diabetic kidney disease (DKD), MEX3C-driven poly-ubiquitination of PTEN promotes EMT, as indicated by enhanced interstitial matrix deposition [27]. MEX3C also influences post-transcriptional gene regulation by modulating mRNA deadenylation and subsequent degradation [29]. MEX3C interacts with several components of the CCR4-NOT complex, providing the main cellular (m)RNA deadenylation activity. K6 and/or K63-linked poly-ubiquitination of CNOT7, one of the main catalytic subunits of CCR4-NOT machinery, by MEX3C increases its deadenylation activity and induces the degradation of the MHC-I mRNA HLA-A2 [29]. However, MEX3C was also proposed to regulate the HLA-A2 mRNA also via a 3′UTR-dependent but RING-independent mechanism [31]. This was also proposed for the MEX3B-dependent regulation of HLA-A2 mRNA [32]. Downregulation of major histocompatibility complex class I (MHC-I) reduces anti-tumor immunity and is described in 40–90% of human tumors [33]. In this context, MEX3 proteins, particularly MEX3B and C, could contribute to an immune evasion of tumors (for review, see Ref. [34]). In sum, these findings suggest a link between the RNA binding and E3 ligase activity, but if and how RNA binding influences E3 ligase activity and substrate recognition and to which extend this is associated with specific RNA interactions and the formation of ternary protein-RNA complexes remains to be unraveled.

MEX3C-driven K63-linked ubiquitination of RIG-I promotes RIG-I activity [28]. RIG-I is a cytoplasmatic sensor for RNA viruses and a key mediator of the antiviral, innate immune response, which essentially involves the coordinated activation of different E3 ligases (TRIM4, TRIM25, Riplet and MEX3C) (for review, see Ref. [35]). In contrast to the other so far identified MEX3 ubiquitination targets, the association with/ubiquitination of RIG-I is independent of the N-terminus including the KH-didomain and is mediated via a direct protein-protein interaction, involving the linker region between the KH-didomain and the RING-finger of MEX3C [28]. Recently, evidence was provided that MEX3A, like MEX3C, influences the ubiquitination of RIG-I [22]. Besides its function in innate antiviral immunity and interferon (IFN) production [36], RIG-I also serves as tumor suppressor [37]. Notably, in contrast to MEX3C, the MEX3A-directed ubiquitination of RIG-I induces proteasomal degradation. Presumably, this is due to K48-linked ubiquitination of RIG-I by MEX3A, a mechanism postulated and also shown for RIG-I regulation by E3 ligases RNF125 and RNF122 (for review, see Ref. [38]).

These findings demonstrate that MEX3-mediated ubiquitination of target proteins leads to quite diverse effects, ranging from protein degradation to (indirect) mRNA deadenylation as well as altered protein activity. Importantly, in some cases, the E3 ligase activity of MEX3 proteins appears linked to RNA association. This suggests that RNA binding may promote either the formation of ternary complexes required for E3 ligase substrate engagement or that RNA binding promotes E3 ligase activity without influencing E3 substrate specificity. However, if and how RNA binding and E3 ligase substrate specificity are linked and if co-regulatory factors are involved remains largely elusive. Notably, however, in view of the high conservation of the KH di- as well as the RING domains of the MEX3 paralogs, it appears unlikely that these domains themselves significantly contribute to RNA or protein target specificity of the individual MEX3 paralogs. More likely appears a pivotal role of the barely conserved auxiliary protein regions, as shown for MEX3C induced ubiquitination of RIG-I [28], which contain extended IDR (intrinsically disordered region) characteristics. In this context, one could speculate that these regions mediate association with co-factors, E3 ligase substrates or even contribute to RNA binding. Based on ANCHOR predictions [39] (Figure 1c), these regions could undergo a disorder-to-order transition upon target binding and thus induce structural rearrangements contributing to protein or even RNA association [40,41,42].

## 4. Roles of Oncofetal MEX3 Proteins in Driving Tumorigenesis

Gene expression data available through the Human Protein Atlas suggest a substantially diverse pattern of expression for human MEX3 paralogs [43]. Whereas MEX3A and B are predominantly expressed in reproductive tissues (e.g., testis, ovary, and endometrium), MEX3C and MEX3D exhibit low overall tissue specificity. Obvious discrepancies between reported protein and mRNA expression as well as publicly available data are discussed in [34].

**Figure 2:**
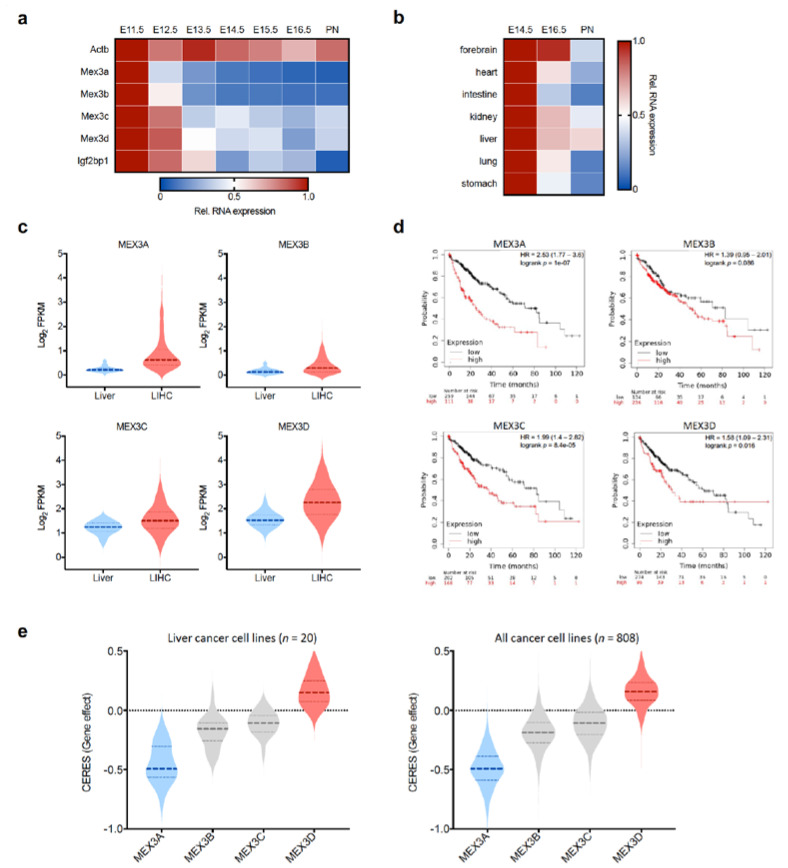
Oncofetal expression of MEX3 proteins. (**a**) The heat map shows the expression of the MEX3 mRNAs during mouse liver development. Actb and Igf2bp1 serve as controls. (**b**) The heat map depicts the expression of MEX3A mRNA during mouse development in various organs. (**a**), (**b**) RNA seq expression data were obtained via the encode data portal (www.encodeproject.org; accessed on 12 March 2021) [44]. (**c**) Upregulation of human MEX3A-D mRNA expression in liver hepatocellular carcinoma (LIHC; *n* = 371; red) versus “normal” liver tissue (blue; *n* = 50) (transcriptome data provided by based on TCGA). (**d**) Survival analysis of MEX3 expression in liver hepatocellular carcinoma. The number of samples considered by kmplot (www.kmplot.com; accessed on 26 March 2021) [47] in the best cutoff and multivariate analyses are indicated. HR, hazard ratio. (**e**). CERES corrected essentiality scores [48] of human MEX3 paralogs based on depmap portal data (www.depmap.org; Accessed on 26 March 2021) for liver cancer cell lines and cell lines from different cancer origins.

Analyses of murine transcriptomics data deposited at the ENCODE data portal (www.encodeproject.org; accessed on 12 March 2021 [44]) suggest that all MEX3 paralogs exhibit an oncofetal expression pattern similar to the oncofetal RBP Igf2bp1. This is characterized by high expression during embryogenesis, decreasing towards birth (Figure 2a). Notably, this oncofetal pattern appears most prominent for MEX3A and B. Consistently, a pivotal role of MEX-3 proteins during embryogenesis/development was provided by knockout studies. In *C. elegans*, MEX3 deletion induces maternal-effect embryonic lethality. MEX3 was proposed to modulate anterior/posterior asymmetry in early-stage embryos by affecting spatially regulated pal-1 expression and appears to be involved in sustaining totipotency within the germline [8,45,46].

Knockout studies of the mouse orthologs MEX3A and MEX3C also reveal a putative impact on development/embryogenesis, whereas MEX3B^-/-^ mice exhibit normal growth and development [49]. Homozygous MEX3C gene-trapped animals were born at Mendelian ratios, and they exhibit postnatal growth retardation and a mouse background-dependent postnatal lethality, presumably via a MEX3C mediated (post)translational control of Igf1 expression [50]. A similar phenotype is described for MEX3A knockout mice. However, MEX3A^-/-^ animals are not born at the Mendelian ratios (nearly 50% die before birth) and also show growth retardation that manifests in weight loss, dehydration and lethargy, leading in up to 60% of newborn to death (P15 to P20) due to alterations in the intestinal tract. Accordingly, MEX3A KO animals exhibit an altered crypt-villus architecture mediated by MEX3A’s function in maintaining an Lgr5+ intestinal stem cell pool presumably by affecting PPARg signaling [51]. Pereira and colleagues did not exclude apoptosis as a putative cause for Lgr5+ stem cell loss due to MEX3A knockout. In accordance with the impact of MEX3A on the intestine epithelium, MEX3A influences a noncanonical, nuclear function of miR-126-5p in maintaining and protecting epithelial cell integrity in the context of autophagy. Deletion of MEX3A in mice abates nuclear accumulation of an Ago2-miR126-5p complex and thereby increases caspase3 activation [13], which in turn could lead to increased apoptosis.

A potential function of MEX3 proteins, especially MEX3 proteins in early development, is supported by their expression in mouse embryonic stem cells [52] and ENCODE-provided mouse transcriptome data. All Mex proteins are highly abundant at early embryonic stages and show a declining expression towards birth (Figure 2a, here shown for liver), being most prominent for MEX3A and MEX3B, in particular in the intestine, stomach and lung tissue (Figure 2b, shown for MEX3A). So far, little is known about the transcriptional control of MEX3 expression. In the human breast cancer cell line MCF10DCIS.com, MEX3A and MEX3B could be identified among the 25 most upregulated targets due to SOX11 overexpression [53]. Sox11 belongs to the SOXC group of transcription factors highly expressed during early embryogenesis and presenting one essential embryonic mammary epithelial marker upregulated in various cancers [54].

Deregulated, mainly upregulated expression of MEX3A-C proteins has been reported in various cancer types (for review, see Ref. [34]); however, the most severe deregulation was observed for MEX3A and MEX3C. Consistently, analyses of, e.g., liver transcriptome data from the TCGA project (www.cancer.gov/tcga; accessed on 26 March 2021) (obtained via the GDC data portal; [55]) revealed a significant upregulation of MEX3 proteins (Figure 2c). This deregulated expression, in most cases, is associated with an overall reduced survival probability suggesting MEX3 proteins, in particular, MEX3A and MEX3C, to serve pro-oncogenic roles (Figure 2d, shown for liver). CRISPR/Cas9-based screens to identify essential genes for survival and proliferation of cancer cell lines analyzed via the depmap portal (www.depmap.org; Accessed on 26 March 2021) corroborates a fundamental function of MEX3A in tumor cell survival (Figure 2e) and suggests MEX3A as a common essential gene in liver cancer cell lines as well as in cell lines from different cancer origins. In contrast to MEX3A, the other MEX3 paralogs have, according to these studies, no significant impact on cell proliferation, whereat MEX3D even exhibits moderate anti-proliferative features as indicated by a computational corrected CERES (gene effect) [48] value above 0.

In conclusion, these findings provide strong evidence that MEX3 proteins, in particular MEX3A, are bona fide oncofetal proteins with roles in specified stem cell niches. As MEX3A promotes proliferation of the intestinal stem cell pool and balances between stem cell self-renewal and differentiation, one could speculate that MEX3A represents a stem cell marker or is involved in the maintenance of stem cell pools per se. Accordingly, ectopic MEX3A expression could enforce proliferation and thus promote cell vitality in cancer and serve as a marker of dismal disease outcome.

## 5. A Snapshot View of MEX3A’s Disease Driving Potential in Cancer

The mechanism underlying the sometimes severe upregulation of MEX3 protein expression in cancer remains largely elusive. However, in mouse models of breast cancer, MEX3A upregulation was induced by SOX11 [53], a member of the SOXC transcription factors, consisting of SOX4, SOX11 and SOX12. Next to functions in neuronal development [56], especially SOX4 and SOX11 were reported to harbor substantial oncogenic potential in various malignancies [57]. Correspondingly, they may serve as transcriptional drivers of MEX3A expression.

Despite epigenetic alterations like altered methylation pattern, as proposed for MEX3A in the context of breast cancer [58], genomic instability events come progressively in the focus of cancer biology, as chromosomal alterations are prominent in nearly all cancers and serve as the hallmark of cancer [59,60]. Analyses of somatic copy number alterations (SCNAs) revealed that roughly 94% harbor an allelic imbalance. In this context, amplification of chromosome 1q was amongst recurrent events in 33 tumor types [60], including hepatocellular carcinoma with a frequency of 46–86% [61]. MEX3A is encoded on chromosome 1q22 within a region (1q21-1q25) that is gained in various cancers [62,63,64] and encodes several pro-oncogenic genes, e.g., RAB25 [65]. In Wilms tumor 1q21.1-q23.2 gain associated with relapse harbors several genes that were also found to be overexpressed, including MEX3A [66]. One could speculate that chromosomal gain of 1q triggers (re-)expression of the oncofetal MEX3A in various cancer entities.

Recently several studies analyzed the function of MEX3A in the context of different tumor types (Table 3), demonstrating in vitro functions of MEX3A in promoting cell proliferation and migration, as well as inhibiting apoptosis. In agreement, transcriptome studies revealed elevated expression of MEX3A in various tumors and association with tumor staging, grading [67,68,69,70], as well as overall survival probability. Based on immunohistochemistry and RNA expression data, MEX3A was therefore suggested as a prognostic tumor marker in various studies [68,69,71,72,73].

Analyses of 31 solid tumor transcriptomes, provided by the TCGA Research Network (www.cancer.gov/tcga; accessed on 12 March 2021) and obtained from the GDC data portal [55], revealed about 270 genes that are positively correlated with MEX3A in at least 20 tumor entities (Spearman correlation coefficient R > 0.25 and FDR < 0.05). In contrast, only four genes showed a likewise negative correlation with MEX3A mRNA abundance (Spearman correlation coefficient R < -0.25; FDR < 0.05). Corresponding gene set enrichment analyses using the R-package clusterProfiler [80] and the MSigDB gene sets [81] identified several hallmark gene sets potentially influenced by MEX3A abundance (Figure S2). Most notably, these include cell cycle progression (E2F_Targets, G2M_Checkpoint) and oncogenic MYC-driven gene expression (MYC_Targets). Alterations of the epithelial-mesenchymal transition hallmark are not this consistent and are obviously dependent on the cancer. However, in vitro and mouse xenograft analyses support a potential function of MEX3A in mesenchymal transition. Consistently, MEX3A knockdown studies revealed migratory potential by reducing CDH2, VIM, SNAI1/2 and increasing CDH1 protein levels, and additionally exhibited reduced lung metastasis [75]. This could be facilitated by an altered PTEN function due to K27-linked ubiquitination mediated by MEX3A, as proposed for MEX3C [26], but further mechanistic studies are necessary to exclude a transcriptional or post-transcriptional regulation of EMT processes. Along this line, several reports indicate a role of MEX3A in the activation of the PIK3CA/AKT signaling pathway [67,74,75,82] that is involved in the regulation of, e.g., proliferation, metabolism, transcriptional control or cell migration (for review see [83]). Accordingly, MEX3A induced migratory phenotypes, as well as pronounced mesenchymal expression patterns, could be mediated by the activation of the AKT pathway affecting inter alia SNAI1, SNAI2 and TWIST levels [84], which are the main transcriptional drivers of EMT. Next to influencing metastatic potential, altered AKT signaling could also contribute to tumor proliferation, as observed in xenograft studies [67,69,72,73,75,82]. This can putatively be induced via destabilization of LAMA2 mRNA by MEX3A, as shown for lung adenocarcinoma cells [75]. MEX3A also affects protein as well as mRNA levels of the cytokine CCL2, which in turn activates AKT signaling and thereby negatively regulates TXNIP [73], a regulator of GLUT1 membrane localization [85,86]. Moreover, MEX3A affects cell cycle progression by stabilizing CDK6 mRNA [68,69], contributing to enhanced proliferation.

Additionally, pro-proliferative and anti-apoptotic effects could be linked to a MEX3A mediated activation of the MAPK/ERK pathway, as shown for colorectal cancer cells [72]. So far, further mechanistic studies, how MEX3A affects these pro-oncogenic signaling pathways are still obsolete.

Despite obviously activating signaling pathways, MEX3A affects several tumor-associated genes, presumably, post-transcriptionally. MEX3A regulates translation of the Caudal-type homeodomain transcription factors 2 (CDX2) by binding to an MRE element in the 3´-utr [51]. The expression of CDX2 in adults is restricted to the gastrointestinal tract [87] and delineates a putative tumor-suppressor and prognostic marker in colorectal cancer [88,89]. Accordingly, increased MEX3A levels could contribute to the repression of the bona fide tumor-suppressive CDX2 in the intestine. Additionally, MEX3A may affect intestinal differentiation by promoting the expression of stem cell markers like BMI1, MSI1 or LGR5 [90].

## 6. Conclusions

MEX3 proteins exhibit pro-oncogenic characteristics presumably mediated by their post-transcriptional and post-translational control of gene expression. Therefore, they influence cell proliferation, migration, signaling pathways and potentially contribute to tumor immune escape (for review, see Ref. [34]).

Within the last months, several reports analyzing the function of MEX3A in carcinogenesis have been published. This increased the knowledge of how MEX3A influences oncogenic phenotypes in vitro and in xenograft studies, but particularly detailed mechanistic studies of post-transcriptional gene control are partially lacking. Although the number of genes affected by MEX3 protein increases, the fewest reports describe or discriminate between post-transcriptional and post-translational control. Along this line, to the best of our knowledge, publicly available data to identify putative mRNA targets and their respective binding sites are lacking as well as putative target proteins that are ubiquitinated by MEX3 proteins. These analyses would help to identify a broader spectrum of MEX3 affected signaling pathways and to shed light on perhaps paralog-specific functions.

## Figures and Tables

**Table 1:** E3 Ligase domain containing RBPs.

Gene Symbol	Protein Name	Domain
AFF4	AF4/FMR2 family member 4	UBOX
ARIH2	ariadne RBR E3 ubiquitin protein ligase 2	RING
BARD1	BRCA1 associated RING domain 1	RING
BRCA1	breast cancer 1, early onset	RING
CNOT4	CCR4-NOT transcription complex, subunit 4	RING
DZIP3	DAZ interacting zinc finger protein 3	RING
MEX3A	RNA-binding protein MEX3A	RING
MEX3B	RNA-binding protein MEX3B	RING
MEX3C	RNA-binding protein MEX3C	RING
MEX3D	RNA-binding protein MEX3D	RING
MID1	midline 1	RING
MKRN1	makorin ring finger protein 1	RING
MKRN2	makorin ring finger protein 2	RING
MKRN3	makorin ring finger protein 3	RING
NFX1	transcriptional repressor NF-X1 isoform 3	RING
PHRF1	PHD and RING finger domain-containing protein 1 isoform 1	RING
PRPF19	pre-mRNA processing factor 19	UBOX
RBBP6	Retinoblastoma-binding protein 6	RING
RC3H1	ring finger and CCCH-type domains 1	RING
RC3H2	ring finger and CCCH-type domains 2	RING
RNF113A	RING finger protein 113A	RING
RNF113B	RING finger protein 113B	RING
RNF17	RING finger protein 17 isoform 2	RING
SCAF11	protein SCAF11	RING
TRIM21	tripartite motif containing 21	RING
TRIM25	tripartite motif containing 25	RING
TRIM40	tripartite motif-containing protein 40 isoform a	RING
TRIM56	tripartite motif containing 56	RING
TRIM71	tripartite motif containing 71, E3 ubiquitin protein ligase	RING
UNK	RING finger protein unkempt homolog	RING
UNKL	unkempt family zinc finger-like	RING
ZNF598	zinc finger protein 598	RING

**Table 2:** Ubiquitination targets of MEX3 paralogs.

MEX3 Paralog	Target Protein	Involved Scaffold	Function	Reference
MEX3A	RIG-I		degradation	[22]
MEX3B	Snurportin-1	lncRNA HOTAIR	degradation	[23]
MEX3B	SUZ12	lncRNA HOTAIR	degradation	[24]
MEX3B	RUNX3	lncRNA HOTAIR	degradation	[25]
MEX3C	PTEN	lncRNA GAEA	switch in activity	[26,27]
MEX3C	RIG-I		activation	[28]
MEX3C	CNOT7		activation	[29]

**Table 3:** MEX3A upregulation in human cancer.

Cancer	Method	Reference
Colorectal	RNA seq.^1^; IHC	[72]
Brain tumors	RNA seq.^1^; IHC	[73]
Pancreatic	RNA seq.^1^; IHC	[68]
Esophageal	RNA seq.^1^; IHC	[69]
Breast	RNA seq.^1^; IHC	[53,58,71,74]
Lung	RNA seq.^1^; IHC	[75]
Liver	RNA seq.^1^	[70,76]
Gastric	qRT-PCR	[77]
Bladder	RNA seq.^1^	[78,79]
Wilms tumor	array-CGH	[66]

^1^ MEX3A mRNA expression analyzed using TCGA provided RNA sequencing data.

## Supplementary Materials

The following are available online at https://www.mdpi.com/article/10.3390/biology10050415/s1, Figure S1: Alignment of MEX3 paralogs’ region spacing KH and RING domains using the “Praline multiple sequence alignment” tool (www.ibi.vu.nl; accessed on 25 September 2020). Figure S2: Heatmap showing normalized enrichment scores (NES) obtained from gene set enrichment analyses (GSEA) of the 50 MSigDB hallmark gene sets based on Spearman correlation coefficients between MEX3A and protein-coding genes in 31 solid tumor cohorts provided by the TCGA.
