# Supplementary material for: Oncogenic Potential of the Dual-Function Protein MEX3A
Source: Biology (Basel). 2021 May 7;10(5):415. doi: 10.3390/biology10050415 (PMC8151450; doi:10.3390/biology10050415)
Supplement: Supplementary file 1 [file biology-10-00415-s001.zip › biology-1195635-supplementary.pdf]

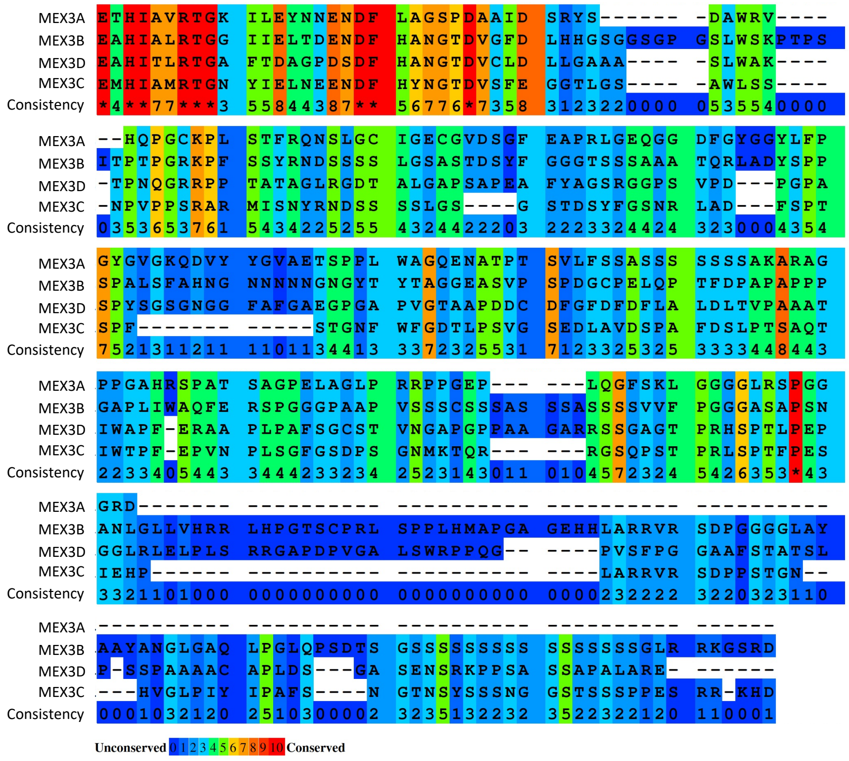

Figure S1: Alignment of MEX3 paralogs' region spacing KH and RING domains using the "Praline multiple sequence alignment" tool (www.ibi.vu.nl; accessed on 25 September 2020). Alignment includes MEX3A (aa285-468), MEX3B (aa222-517), MEX3D (aa335-599) and MEX3C (aa388-607).

Supplementary Figure 1

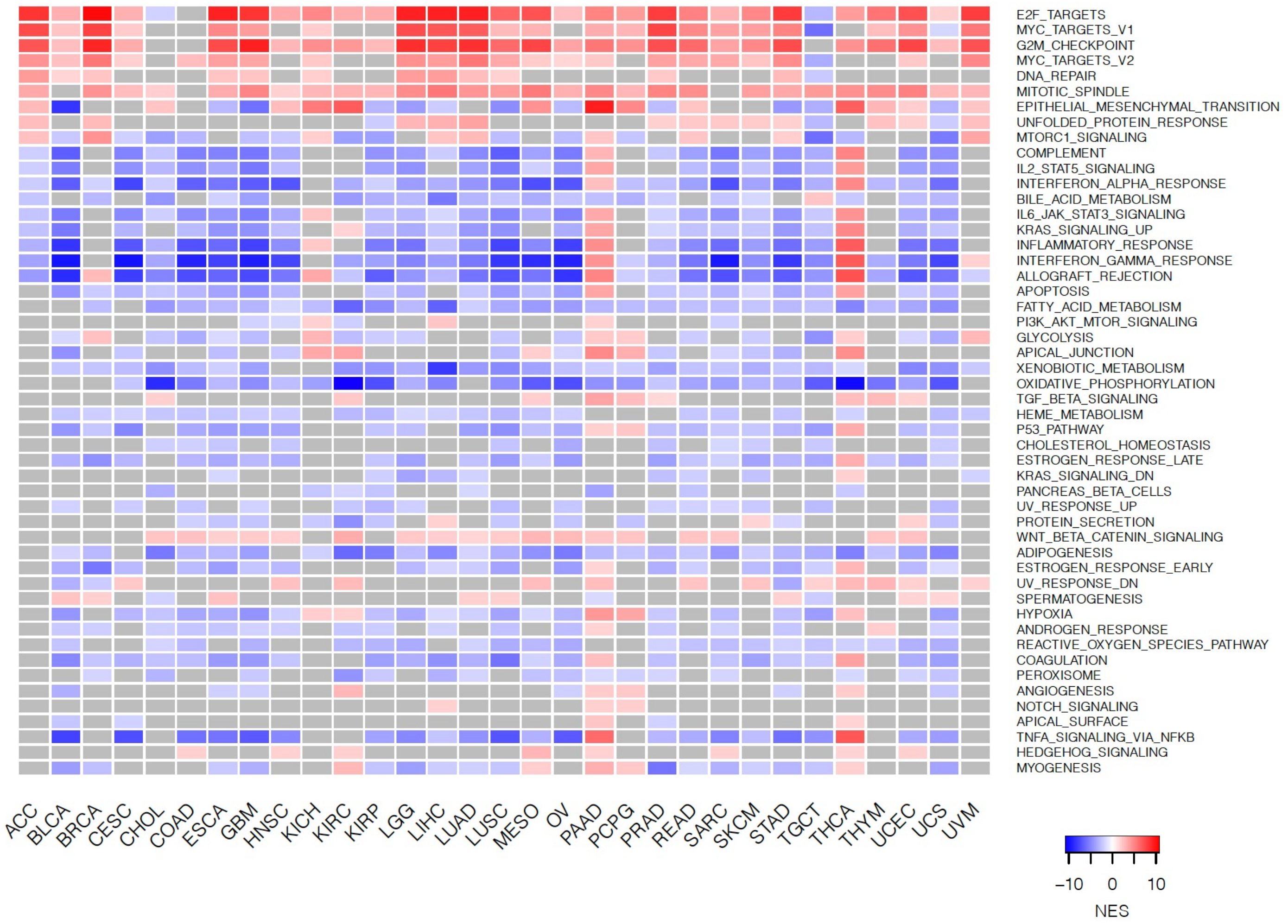

Figure S2: Heatmap showing normalized enrichment scores (NES) obtained from gene set enrichment analyses (GSEA) of the 50 MSigDB hallmark gene sets based on Spearman correlation coefficients between MEX3A and protein-coding genes in 31 solid tumor cohorts provided by the TCGA. Gray values represent non-significant results. Abbreviations according to TCGA cancer group acronyms.

Supplementary Figure 2
